# Patient access to complex chronic disease records on the Internet

**DOI:** 10.1186/1472-6947-12-87

**Published:** 2012-08-06

**Authors:** Cherry Bartlett, Keith Simpson, A Neil Turner

**Affiliations:** 1 Department Health Sciences, University of York, York, YO10 5ZZ, UK; 2 Renal Medicine, Royal Infirmary, Glasgow, G4 0SF, UK; 3 Renal Medicine, University of Edinburgh, Royal Infirmary, Edinburgh, EH16 4SA, UK

## Abstract

**Background:**

Access to medical records on the Internet has been reported to be acceptable and popular with patients, although most published evaluations have been of primary care or office-based practice. We tested the feasibility and acceptability of making unscreened results and data from a complex chronic disease pathway (renal medicine) available to patients over the Internet in a project involving more than half of renal units in the UK.

**Methods:**

Content and presentation of the Renal PatientView (RPV) system was developed with patient groups. It was designed to receive information from multiple local information systems and to require minimal extra work in units. After piloting in 4 centres in 2005 it was made available more widely. Opinions were sought from both patients who enrolled and from those who did not in a paper survey, and from staff in an electronic survey. Anonymous data on enrolments and usage were extracted from the webserver.

**Results:**

By mid 2011 over 17,000 patients from 47 of the 75 renal units in the UK had registered. Users had a wide age range (<10 to >90 yrs) but were younger and had more years of education than non-users. They were enthusiastic about the concept, found it easy to use, and 80% felt it gave them a better understanding of their disease. The most common reason for not enrolling was being unaware of the system. A minority of patients had security concerns, and these were reduced after enrolling.

Staff responses were also strongly positive. They reported that it aided patient concordance and disease management, and increased the quality of consultations with a neutral effect on consultation length. Neither patient nor staff responses suggested that RPV led to an overall increase in patient anxiety or to an increased burden on renal units beyond the time required to enrol each patient.

**Conclusions:**

Patient Internet access to secondary care records concerning a complex chronic disease is feasible and popular, providing an increased sense of empowerment and understanding, with no serious identified negative consequences. Security concerns were present but rarely prevented participation. These are powerful reasons to make this type of access more widely available.

## Background

Patient access to their healthcare records is a legal right in many countries, and access via the Internet is a stated aim of government policy in the UK and elsewhere, although slow progress has been made in achieving it. Many published evaluations have involved primary care or outpatient (office-based) practice. In general, patients welcome access to their written records and prior concerns of clinicians have been reduced by practical experience[1,2]. In wider discussion of patient access to records and to health information on the Internet, quality of information is a recurrent concern and a stimulus to developing such systems, while uncertainties about impact on current ways of working are a potential barrier to implementation[3].

Renal Medicine is a numbers-intensive specialty with a reputation for complexity. It pioneered some of the first electronic patient record systems in the world[4,5]. In 2004 a gathering of UK patients and professional organisations meeting as the Renal Information Exchange Group (RIXG, http://www.renal.org/rixg) discussed how to exploit advances in IT to improve patient experience. Their top priority was to make it possible for patients to view major parts of electronic patient records via the Internet.

We aimed to produce a system that would couple presentation of timely clinical data with links to information to explain test results, diagnoses and treatment, so far as possible utilising information already stored electronically. It was designed to be easy to set up and low-maintenance to run. Renal PatientView (RPV) has been enthusiastically taken up in the majority of UK renal centres and continues to spread.

## Methods

The content of the online record was based on patient questionnaires and feedback from focus groups, shaped by interactions with clinicians and renal IT specialists working in renal units. Small external companies were commissioned to develop the system. Development work, testing and security policies were overseen by a small steering group of clinicians and IT professionals.

### System development

An initial system with demographic data and a limited set of results was tested by 10 volunteer patients before going live in 4 pilot units in 2005. Results shown were 10 key items identified by patients including tests of kidney function and anaemia. After feedback the project was broadened and the system made available to any renal centre where the necessary data could be extracted from local systems. Information links to be associated with specific diagnoses and treatments were selected by an informal group of clinicians and patients from RIXG and more widely. These links can be easily added to or altered without requiring a specialist developer. Further information and test logins are linked from the home page at http://www.renalpatientview.org.

Local renal IT systems are configured to send any new data at a set interval (usually once or twice daily) for each enrolled patient. Results are transferred automatically without prescreening, so that patients may on occasion read bad news (such as deterioration in kidney function) or even dangerous results (such as high potassium) before their clinicians.

Data is transmitted as an encrypted XML file and all other communications with the webserver are encrypted. Records are based on a unique patient identifier (NHS number, or CHI number in Scotland), enabling a composite record to be generated when data is received from more than one location, such as from the base renal unit and from a transplant unit. Security and confidentiality procedures were developed in accordance with relevant National Health Service (NHS) policies and the UK Data Protection Act [6]. Server security was verified by external specialists.

**Figure 1:**
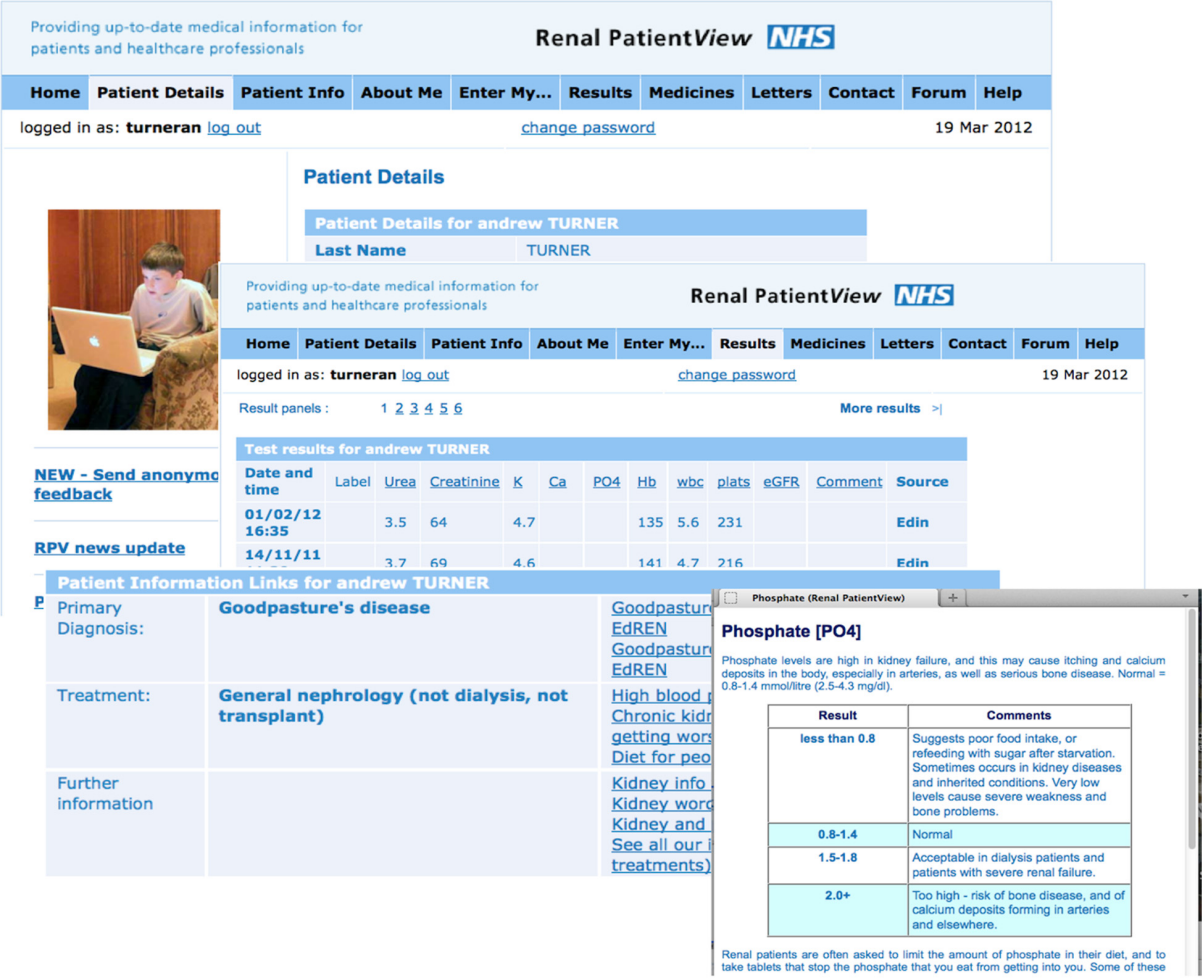
Renal PatientView screenshots.

Examples of the data shown are given in Additional file 1 and Figure 1. All data comes from the local renal electronic patient record (EPR) except for Transplant list status, which is the definitive status reported from UK Transplant, as specifically requested by patients following reported communication failures. At the time of this research the system was ‘read only’; it did not include any interactive features or the ability to communicate directly with clinicians, or to view or arrange dates of appointments.

Patients learn about the system from leaflets and posters or by recommendation in participating units. Enrolment involves signing a request form that is countersigned by a member of staff who can verify the patient’s identity. A local renal unit administrator initiates regular sending of encrypted data from the local renal EPR, and generates a login from the webserver. Logins are sent to the patient’s registered address.

Modest installation and running costs are recovered from participating renal units. There is no charge to patients and there is no financial benefit from participation. Patient care (including number of visits, tests) is not influenced by using the system.

### Evaluation

Self-administered questionnaires (Appendix 1) were developed for users (registrants) and non-users and sent to all patients registered as users at Leeds, Heart of England (Birmingham), York, and Edinburgh renal units, and to a random sample of non-users. Responses from carers were accepted. Follow-up copies of the same questionnaire were sent to all non-responders and to Glasgow Western Infirmary users and non-users to increase numbers. A brief message was displayed on the website to encourage participation. The questionnaires included a predetermined set of questions and space for comment on any aspects of the website and the system.

Staff in units using RPV were invited by email to complete and send on to others in their unit a link to an online survey seeking their views on the system and its effects on patients and on their work. In addition to questions on effects on patients it included questions on whether there had been any change in workload and each question included areas for free text comments. Both surveys were completed by the end of 2008.

Enrolment numbers were determined from server records. Simple usage analysis was available from Google Analytics data.

## Results

### System development

Experience from pilot units supported the value of the system, with strongly positive informal feedback from patients and no substantial problems encountered by staff.

We chose to pilot viewing of unscreened (unapproved, live) results because of concerns about the practicality of putting an extra task on hard pressed clinicians and the inevitable delays that this would introduce in presenting the results to patients. Concerns about this turned out to be unfounded. Patients are anxious to know results and it seems they would rather have bad news sooner than wait for clinicians to deliver it. Anecdotally, they were not convinced that bad news was made less bad by being delivered by a member of staff. A clinician commented ‘if a patient contacts the unit about a dangerous potassium value before we have contacted them, that is a good thing’. In the light of this experience, we continued the practice of releasing live data as the project extended.

### Patients and patient evaluation

**Figure 2:**
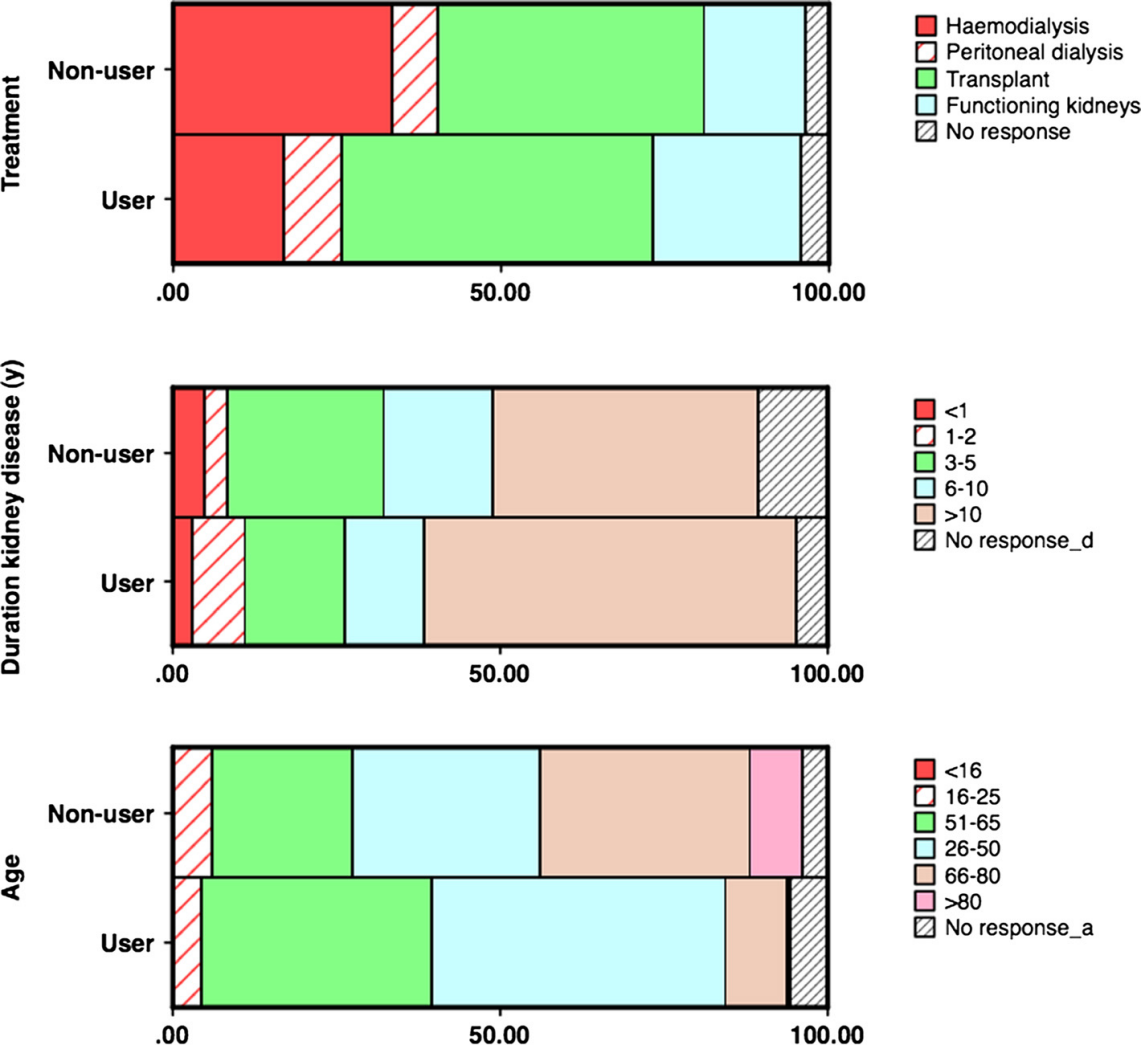
Proportions of registrants (users) and non-registrants by age, treatment modality and duration of renal follow up (%) in the evaluation survey.

499 patients who had registered to use Renal PatientView (63% of recipients) and 84 who had not registered (43%) returned completed questionnaires. Of user responses, 93% were from patients, 6% carers, 1% parents. Registrants were younger (median 50y versus 57y), but the age-range of patients enrolled was from young children to over 90 years (no paediatric units were included in the evaluation survey). The sex ratio was not significantly different from that of non-registrants. Some differences between registrants and non- registrants hint at the factors that drive patients to seek information about their health: registrants were more likely to have transplants or functioning (likely deteriorating) kidneys, and less likely to be on dialysis; moreover, users were more likely to have had their kidney problem for longer periods, especially longer than 6 years (Table 1 and Figure 2). It is likely that the internet-based approach was more accessible to more educated as well as younger patient groups as the age of leaving full-time education was older in registrants than in non- registrants in the patient survey (median 17-18y versus ≤16y).

**Table 1:** Characteristics of patients participating in the evaluation survey

	**Users % (n = 499)**	**Non-users % (n = 84)**
Gender		
Male	54.5	54.8
Age		
<16	0	0
16-25	4.4	6
26-50	47.7	28.6
51-65	36.3	21.4
66-80	9.8	32.1
>80	0.4	8
No response	5.8	3.9
Duration kidney disease (y)		
<1	3	4.8
1-2	8	3.6
3-5	15.2	23.8
6-10	12.2	16.7
>10	56.9	40.5
No response	4.7	10.6
Treatment		
Haemodialysis	16.8	33.3
Peritoneal dialysis	8.8	7.1
Transplant	47.5	40.5
Functioning kidneys	22.6	15.5
No response	4.3	3.6
Ethnicity		
White British	93.2	90.5

At the time of the survey respondents reported using the site for a mean of 7.2 months. 88% had accessed it more than twice and 32% over 10 times. 5% had not accessed it at all. The dominant reason given by non-users for not being registered was lack of knowledge of the system (58%), followed by not being able to access the Internet.

Users found the site easy to access and use, and reported the information available to be valuable or very valuable, and that the contents gave them better understanding and empowerment. The results section was the most frequently accessed area. 24% acknowledged ‘I read things that worry me’.

Security was not a matter of concern to the majority of users, but 26% reported being slightly and 7% ‘very concerned’ before enrolling. The level of concern was reduced after using the system, 12% reported being slightly and 1% very concerned. 18% of patients reported that they had changed their passwords after the first forced change.

The extra information most desired by patients was clinical correspondence, which was not available in most of the test units at the time of the survey. There were multiple requests for additional test types, and to see the results of tests sent from other healthcare locations. Written comments reinforced the numerical feedback (Additional file 1).

By the end of June 2011, 17,473 registrations were recorded from 47 of the UK’s 75 main renal units (63%). Numbers of patients enrolled in each unit varied from tens to 1,340. Looking at units that had RPV installed for more than 18–24 months there was no clear relationship between length of time that the system had been installed and the registration rate.

Analysis of actual usage data confirms patients’ reports that the system is valued and used. During a typical month (figures are for June 2011) there were an average of 859 visits per day. 9391 registrants, over half of the total, logged in in a single month. Each of these visited the site a mean of 2.8 times during the month. Average visit length was 4.54 minutes and a mean of 9 pages were viewed.

### Staff evaluation

99 staff members from at least 31 centres (centre not given by 22 respondents) responded to an email requesting feedback. It was not possible to record the response rate as recipients were invited to forward the survey to staff who were aware of the system. Responders included doctors (51%), nurses (27%), and renal IT professionals (11%), secretarial and administrative staff (11%).

Strikingly, staff ratings of the system were as positive as those from patients. 82% felt that it had altered patient care for the better, 0% for the worse. 74% felt that it had improved patients’ confidence in their care. 95% felt that it had no effect on the length of consultations.

In general use of the system, the most frequent reason for contacting system administrators was a forgotten login, followed by contacts to amend demographic information or errors in data shown. These have including correcting contact numbers for patients on the Transplant list, information about drugs prescribed, and observations on clinically important matters such as potassium levels, dialysis adequacy. There were some reports that minor fluctuations in creatinine or eGFR had led to unnecessary concern. There were no reports of generally increased patient anxiety and most units reported reductions in telephone inquiries, but the numbers of telephone calls or other enquiries were not measured formally.

## Discussion

This is the first national project in the UK to offer Internet access to secondary care (hospital) records for a chronic disease, and unusual internationally in giving access to Renal records. Some of our experience in this more than usually results-intense specialty has been similar to other evaluations of patients’ access to records, in that user responses have been strongly positive. Almost all reported studies describe positive benefits for patients’ perceptions and/or empowerment, with no apparent significant negative outcomes[7,8]. Most are observational studies but a cluster-randomized study in primary care concurred[7,9].

### Profile of users

The small difference in age between users and non-users, and lack of sex difference, are surprising. Health care information is widely sought on the Internet, particularly by patients with chronic diseases[10-12], but most surveys have identified a female preponderance, as also seen in attendances for medical consultation[13,14].

As in other studies of specific portals as well as more general surveys of healthcare-related Internet use [15-17], our users were younger than non-users, but the difference was relatively small and the age range of users was wide. Internet use continues to rise rapidly in the UK (67% of adults and 61% of households in 2007, rising to 84% and 77% by 2012[18]), and in some units over half of those receiving renal replacement therapy have enrolled, suggesting that potential penetration of the system overall is much higher than achieved at present. This may also suggest that any current differences in usage by different demographics could reduce progressively.

An effect of social disadvantage or income, in which disadvantaged individuals are less likely to look up health information on the Internet, has been mentioned in some previous studies. Lobach et al.[19] found that less education and lower income did not necessarily mean less desire to access records, even where it affected uptake. The reduced educational experience of non-users in our user survey suggests that opportunity or educational achievement may influence uptake, and further investigation of the potential effect of social factors will be important.

### Uptake and usage

Looking up health-related information about others on the Internet is common[11 12]. We did not quantitate how many enrolled patients relied on another family member for access to RPV, but a small survey in a single unit (Reading; McGlashan, personal communication) did ask that question. Of 67 patients, 28% allowed someone else to use RPV on their behalf, while 15% of users never used the system themselves and always relied on someone else logging in. Login sharing could be reducing the differences between ages and groups that we might otherwise have observed in RPV users.

Comparison of patient registration numbers with size of dialysis/transplant populations cared for by units suggests that in several units enrolment in this patient group is approaching or in excess of 50%. The relatively high uptake and usage of Renal PatientView may be a consequence of patients having significant long-term disease, or it could be something specific to renal disease. It will be interesting to investigate it further as it suggests that the barriers to using the Internet more widely in this way could be lower than previously feared, if the circumstances are right. ‘Circumstances’ are likely to include the perceived value of the information being shown (or the interactions offered), and the accessibility and usability of the system delivering it.

Uptake between renal units varied widely. The patient survey suggested that even in the enthusiastic early-adopter units that participated in the survey, the main reason patients gave for not enrolling with RPV was lack of awareness of the system. Initially we observed that many staff were cautious about encouraging uptake for fear that it might increase their work, or make their lives more difficult. The staff survey confirmed that this was not their experience, and we predicted that this would gradually lead to wider recommendation. However independent surveys of dialysis and transplant patients undertaken in Scotland in 2009 revealed continuing high levels of unawareness of Renal PatientView, even in units with high levels of patient enrolment. 33-62% of dialysis patients and 60-78% of transplant patients said that they were aware of the system in Scottish units in which it was available[20]. From anecdotal reports, we believe that a significant difference between high-enrolling and low-enrolling units is likely to be the frequency with which it is advocated by staff. Even when there are many posters and leaflets in circulation, patients do not always perceive that it is available to them or that it might be useful for them until it is specifically recommended.

### Responses to records access

It is difficult to prove that patient access to records results in improved healthcare outcomes. As the introduction of such a system is generally accompanied by multiple other changes made by enthusiasts this is difficult to study in a well controlled manner. However it has been pointed out that such a demanding proof of effectiveness of online services is not generally felt necessary in other industries where clients/customers benefit from better access, such as banking and travel. There is however some evidence that patient concordance can be improved by access to records [21], and chronic renal disease is an area where adherence to therapy is likely to bring about improved outcomes. Our experience suggests that patient access improved the accuracy of electronic records as patient-directed corrections were common.

It is interesting that some patients enrolled in Renal PatientView despite significant prior concerns about security and confidentiality. Presumably they rated the value of access to their data highly enough to outweigh these concerns. There is evidence from a theoretical examination of how patients would feel about online records that concern reduces as disease burden rises[22]. Both lines of evidence suggest that patients assess their personal risk/benefit ratio.

We were initially concerned that some patients reported ‘I read things that worry me’, but on reflection this is less surprising. Chronic renal disease is a serious, lifelong condition that critically affects the lives of renal patients, and concern about this is likely to be important in explaining why patients want to use the system. If users were not concerned, or considering the possibility of bad news, would the system be so valuable to them?

Our experience with Renal PatientView suggests that it is frequently accessed, valued, has dominantly positive effects, and that such systems can be introduced at low additional cost while requiring minimal extra input from clinicians. It will be interesting to see how far its use can penetrate, and to further characterise what are the barriers to using it.

It would be relatively easy to extend this type of information provision to other specialties with existing electronically held patient data. In the long term, integration with information from primary care records and held by other specialties must be the aim.

## Conclusions

Patient internet access to secondary care records concerning a complex chronic disease is feasible and popular, providing an increased sense of empowerment and understanding, with no serious identified negative consequences.

## Competing interests

The author(s) declare that they have no competing interests.

## Authors’ contributions

CB, KS and NT formed the nucleus of the project design and steering group, and wrote the paper. CB designed and analysed the evaluation. All authors read and approved the final manuscript.

## Pre-publication history

The pre-publication history for this paper can be accessed here:

http://www.biomedcentral.com/1472-6947/12/87/prepub

## Supplementary Material

Additional file 1 Content of Renal PatientView record. Click here for file
