# Supplementary material for: Patient access to complex chronic disease records on the Internet
Source: BMC Med Inform Decis Mak. 2012 Aug 6;12:87. doi: 10.1186/1472-6947-12-87 (PMC3438097; doi:10.1186/1472-6947-12-87)
Supplement: Additional file 1 — Content of Renal PatientView record. [file 1472-6947-12-87-S1.doc]

**Box 1**

**Content of Renal PatientView record**

*Patient details*

- Demographic and contact info
- Renal diagnosis (coded; EDTA codes already in use)
- Treatment (coded; haemodialysis, peritoneal dialysis, transplant, general nephrology)
- Transplant list status (directly from UK Transplant)
- Problem List (free text) if available

*Info links*

- According to diagnosis code
- According to treatment code

*Results –* multiple panels of results of common biochemical and haematological tests including Hb, wbc, plats, urea, creatinine, potassium, sodium, bicarbonate, calcium, phosphate, parathyroid hormone, dialysis adequacy, eGFR, levels of immunosuppressant drugs. With links to an explanation of the test and its expected values/targets.

*Letters -* If stored on unit’s IT system

*Medicines -* If stored on unit’s IT system

*Contact info -* For contacting local unit and primary care physician

**Box 2**

**Comments from users**

I felt like a pest keeping ringing the hospital for my results after I had attended clinic – I now feel that my results belong to me.

… one of the best innovations I have encountered in the NHS … in my 50yrs involvement”

Patients don’t always take in what the medics say, and to be able to take time to read it properly in the comfort of your own home is wonderful!

I feel much more relaxed about my transplant when I know my results from my clinic appointment so quickly.

On behalf of my mother, thank you so much for the patient view system” (of patient in her 70s)

I don’t show it to my mum, it would worry her (17 year old patient)

a great service – especially for the ‘yo-yo’ generation who seem never to be in the same place for more than a few days … a lot of stress is removed, and the instant access is great

… it enables me to have a blood test a week before my Consultant’s appointment and see the results – allowing a far more informed discussion of treatment

**Comments from staff**

… non-compliers have become much more involved in their care through looking up results and reading letters online

Confidence-boosting in asking questions about their own care and how they can self help

It has shown some of the details we hold to be incorrect

Several patients improved compliance once they had up to date access to their biochemistry

I expressed some very real concerns about a patient’s suitability for transplant. This led to a frank discussion and clear the air, so in the end beneficial.

Has increased the confidence of patients as they see a more transparent service and feel involved in their own care.

It saves phone calls

It is excellent and improves patient safety

It is a reassurance when going away on holiday

Increases the number of patients who are more engaged with their own care and helps them get more value of their medical consultations

Consultants regularly comment on what a revelation it has been for patient care
